# Supplementary material for: Entanglement-inspired frequency-agile rangefinding
Source: Nat Commun. 2026 Jan 24;17:2001. doi: 10.1038/s41467-026-68589-9 (PMC12936112; doi:10.1038/s41467-026-68589-9)
Supplement: Supplementary file 1 — Supplementary Information [file 41467_2026_68589_MOESM1_ESM.pdf]

Supplementary information: Entanglement-inspired frequency-agile rangefinding

Weijie Nie,[1,*] Peide Zhang,[1] Alex McMillan,[1] Alex S. Clark,[1] and John G. Rarity[1]

[1] *Quantum Engineering Technology Labs, H. H. Wills Physics Laboratory and School of Electrical, Electronic and Mechanical Engineering, University of Bristol, BS8 1FD, Bristol, United Kingdom*

(Dated: January 12, 2026)

I. ENERGY-TIME CORRELATIONS FROM PULSE STRETCHING

The generation of energy–time correlations in the developed source is achieved through fibre-induced chromatic dispersion, followed by time-domain post-selection using an electro-optic intensity modulator (EOIM), as detailed in Methods Online. To elucidate the underlying mechanism, we begin by describing the temporal stretching of the pulsed laser in the dispersive fibre, which is governed by the wave-vector k, as a function of the angular frequency ω, expressed as follows

$$k(\omega) = k(\omega_0) + \beta_1 (\omega - \omega_0) + \frac{1}{2}\beta_2 (\omega - \omega_0)^2 + \ldots \quad \text{(S1)}$$

where ω_0 is the central angular frequency, while β_1 and β_2 describe the group delay and the quadratic group velocity dispersion, respectively. The transmission time T for light with angular frequency ω travelling through a fibre of length L is given by

$$T(\omega) = L\,|\beta_2|\,\omega - L\,|\beta_2|\,\omega_0 + T(\omega_0) \quad \text{(S2)}$$

This expression can be reformulated in terms of photon energy E to describe the energy-time correlation, where the transmission time T as a function of photon energy E is

$$T(E) = L\,|\beta_2|\,E - L\,|\beta_2|\,\frac{E_0}{\hbar} + T\left(\frac{E_0}{\hbar}\right) \quad \text{(S3)}$$

where E_0 is the central photon energy, and $\hbar$ is the reduced Planck constant. By selecting different time delays within each pulse, different energy channels can be accessed.

II. SIGNAL-TO-NOISE RATIO ENHANCEMENT

Building on the generated energy–time correlations in the broadband pulse, the pulses are selectively modulated at distinct time delays and encoded with a pseudo-random time sequence afterwards, enabling frequency agility in the developed source. The resulting pulses in random wavelength channels are transmitted to a target, and reflected photons are collected, separated into different wavelength channels, and sent to single-photon detectors. We then calculate the time-delayed cross-correlation function to find the coincidence rate at different time delays τ

$$C(\tau) = \int_0^{T_t} \langle S(t)R(t+\tau)\rangle dt \quad \text{(S4)}$$

where S is the photon detection signal, R is the normalized known reference signal from the energy-time correlation, and T_t is the time over which the random pulses are sent. The histogram will show a peak corresponding to the time of flight of the reflected photon signal that can then be used to calculate the round-trip range to the target.

To evaluate the noise reduction capability of this entanglement-inspired n-channel source in a rangefinding system, we can calculate the signal-to-noise ratio (SNR) assuming the system is shot-noise limited as

$$\text{SNR} = \frac{\sum_{i=1}^{n} C_{S_i}}{\sqrt{\sum_{i=1}^{n} C_{S_i} + \sum_{i=1}^{n} C_{N_i}}}\,, \quad \text{(S5)}$$

where C_{S_i} represents the signal photon coincidence counts integrated in a time window around the coincidence peak, and C_{N_i} is the average coincidence noise counts during the same period. The return signal for each channel i is

$$C_{S_i} = \frac{N_S}{n}\eta_i\eta_S\,, \quad \text{(S6)}$$

where N_S is the total number of photons emitted by the transmitter spread across all n channels, η_i is the unbalance in channel power splitting with $\sum_{i=1}^{n}\eta_i = n$, and $\eta_S = \eta_t\eta_r\eta_d$ is the total channel efficiency taking into account the transmission and collection efficiency η_t, the reflection of the target η_r and the detector efficiency η_d. We make the assumption here that these are the same for all channels as the difference between each channel is negligible. The primary noise in each channel is

$$C_{N_i} = C_{B_i} + C_{D_i}\,. \quad \text{(S7)}$$

where C_{B_i} is the background count from the environment, such as solar background, C_{D_i} is the dark count from the single-photon detectors. The background count is

$$C_{B_i} = \frac{B_0 \cdot \Delta\lambda}{n}\eta_{ub}\frac{l \cdot T_p}{n}\eta_B = \frac{N_B}{n^2}\eta_{ub}\eta_B\,, \quad \text{(S8)}$$

where N_B is the total number of background photons present when the pulses are on. η_{ub} is the channel unbalance in background counts splitting with $\sum_{ub=1}^{n}\eta_{ub} = 1$,

* weijie.nie@bristol.ac.uk

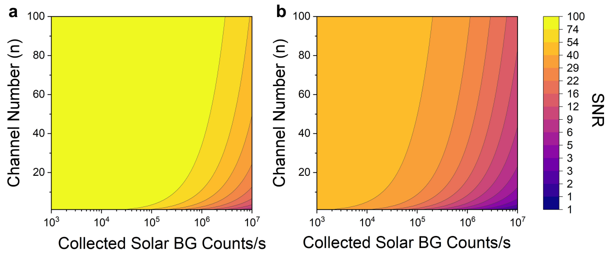

FIG. S1. **Theoretical SNR improvements.** | Simulated SNR under high transmission losses of (a) 98.1 dB and (b) 104.8 dB, corresponding to a distance of 155 metres under sunny and rainy conditions, respectively, as a function of the number of channels and collected solar background (BG) counts per second. All parameters are set to the experimental values.

$\eta_B = \eta_c\eta_d$ is the lumped system efficiency for collecting (η_c) and detecting (η_d) background photons. We can see that N_B is found by multiplying the background spectral density, B_0 in Hz/nm, and the total bandwidth of all energy channels, $\Delta\lambda$, normalised by the number of channels, n. This must then be multiplied by the total on-time of the reference pattern used in the cross-correlation that forms the coincidence histogram. The random pulse pattern has integer length l, pulses are shared between all channels n, and each pulse has a duration T_p. The dark counts from each photon detector are,

$$C_{D_i} = \frac{D}{n}\eta_{ud}\frac{l \cdot T_p}{n} = \frac{N_D}{n^2}\eta_{ud}\,, \tag{S9}$$

where D is the total dark counts of all single photon detectors. η_{ud} is the imbalance in the dark counts of different detectors related to the channel splitting, where $\sum_{ud=1}^{n}\eta_{ud} = 1$. The second term once again takes into account the time that the reference pattern pulses are on for each channel. Similar to background noise, We can write this as the all detectors dark counts when the total pattern is on, N_D, which is divided by a square of channel number n. Substituting Eq. S8, and Eq. S9 into Eq. S7, and substituting that and Eq. S6 into Eq. S5 yields

$$\begin{aligned}\mathrm{SNR} &= \frac{N_S\eta_S}{\sqrt{N_S\eta_S + N_D/n + N_B\eta_B/n}}\\ &= \frac{\mathcal{S}}{\sqrt{\mathcal{S} + \mathcal{D}/n + \mathcal{B}/n}}\end{aligned} \tag{S10}$$

where $\mathcal{S}$, $\mathcal{D}$ and $\mathcal{B}$ are the total correlated signal counts, dark counts and background counts across all channels (i.e., the counts recorded while the reference pattern is on). Upon summation over all channels, all imbalance parameters (η_i, η_{ub}, and η_{ud}) are effectively cancelled. From this theoretical model, two distinct advantages of our approach over traditional quantum rangefinding [1] can be observed. First, the impact of background noise N_B can be mitigated by increasing the number of channels n, allowing for significant noise reduction. Unlike quantum systems, where the optimal number of channels is constrained due to the contribution of dark counts in heralding detectors to the total noise, classical systems can exploit arbitrarily large numbers of channels without such limitations. Therefore, our system allows for SNR improvements as more channels are added. Second, while the number of channels increases, noise due to dark counts remains constant because each detector only contributes counts when the pattern is assigned to that channel. In other words, the dark counts from all detectors only contributes when the time window is correlated to its energy channel, resulting in invariant total dark counts due to the same total time. This ability enables effective scaling of the system without sacrificing performance, making it highly suitable for practical remote sensing applications.

These advantages are clearly illustrated in Fig. S1(a), when the detection range is set to 155 metres – corresponding to our field trial distance with a transmission loss of 98.1 dB in bright sunlight – the SNR is significantly enhanced under high background noise levels (e.g. 3×10^7 Hz detected photon counts cross 100 Single-Photon Avalanche Diode (SPADs)) by increasing the number of energy channels from 1 to 100. Figure S1(b) depicts the increased loss during rainy conditions, demonstrating a more pronounced improvement under high background noise levels. This underscores the robustness of the correlation system in real-world scenarios, highlighting its potential for deployment in environments with elevated ambient light levels.

III. ELECTRICAL SIGNAL PROCESSING

To realize precise energy channel selection, the EOIM is driven by a 250 ps electrical signal with a variable time delay programmed by the FPGA (Xilinx ZYNQ-7000). The original electrical pulse is derived from the femtosecond laser to ensure synchronization, featuring a pulse width of 370 ps and a repetition rate of 100 MHz. The pulses are subsequently broadened to 4 ns by a pulse shaper to be reliably detected by the FPGA input, and are then down-converted to 50 MHz, as constrained by the operating range of the FPGA's IDELAY module. A pseudo-random sequence of variable time delays is generated and encoded by the internal IDELAY module onto 500 consecutive pulses, synchronized with the down-converted 100-kHz reference signal sent to the time-tagger (HydraHarp 400). Finally, the electrical pulses are compressed to 250 ps (to enhance temporal resolution) and amplified to 3 V (to improve the extinction ratio, though still below the full 6-V drive limit due to amplifier saturation). These pulses are then applied to the EOIM, which serves as a high-speed optical switch that randomly selects wavelength channels according to the encoded pattern.

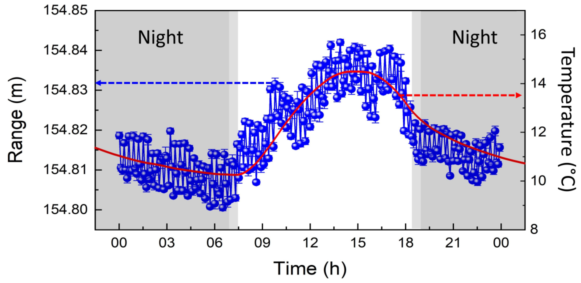

FIG. S2. **24-hour continuous field testing.** | Detected range (Blue) and hourly average temperature (Red) as a function of time over the 24-hour measurement period, from midnight to the following midnight. The range is measured with an integration time of 0.1 s. Error bars represent the standard deviation of 60 independent range measurements acquired within each 1-second time window. The range is found at a sampling interval of 6 min. The hourly average temperature data were recorded from the Bristol Airport weather station from WeatherSpark.com [2]. Nighttime and civil twilight are indicated by shaded background overlays.

IV. LONG-TERM STABILITY AND ENVIRONMENTAL ROBUSTNESS

Considering long-term stability and repeatability, a systematic evaluation of environmental robustness was performed through continuous 24-hour field testing. As shown in Fig. S2, the measured distance was recorded from midnight to the following midnight with an integration time of 0.1 s and a sampling interval of 6 min. Measurement repeatability was determined from 60 measurements acquired within each 1-second interval, represented as error bars in the plot.

A range fluctuation of approximately 11 mm (corresponding to a $\sim$73 ps time drift) with a period of $\sim$24 min was observed, which is likely caused by temperature-dependent delay and phase variations in the Zynq-7000 FPGA's internal clocking and routing circuitry. In future work, this will be addressed by using closed-loop temperature control of the FPGA. The overall long-term range tendency is attributed to thermal effects and follows the recorded temperature evolution, as indicated by the average temperature variation trace (red line in Fig. S2). While such effects can be mitigated using improved thermal isolation of the fibre cable, the inherent temperature sensitivity may also serve as a useful feature for tracking environmental temperature changes. Importantly, the measured distance returns to its initial value at night, confirming strong measurement consistency under fluctuating ambient conditions. Additional factors that may influence system stability are further analysed and discussed below:

Alignment stability: The optical alignment remained stable throughout day-to-day field measurements and was unaffected by changes in lighting or weather. As shown in Fig. S2, all optical components were securely fixed on a rigid mounting platform, and the transmitter and receiver shared the same multi-axis rotation stage, ensuring structural stability. In addition, the use of Lambertian backscattering relaxes alignment constraints, as detection is less sensitive to variations in target surface angle.

Thermal effects: The fibres in the source and detection modules were housed in a temperature-controlled laboratory environment, where temperature-induced variations are minimal. Only the 300 m outdoor telecom fibre linking the laboratory and the balcony was exposed to environmental temperature changes. As shown in Fig. S2, the range change from 3 a.m. to 3 p.m. is approximately 20 mm, corresponding to a 133.3 ps timing difference and an 11.4 K temperature change, with a thermal coefficient of 39 ps$\cdot$km$^{-1}\cdot$K^{-1} [3]. The hourly average temperature data (red line) from the Bristol Airport weather station [2] indicates a temperature variation of about 4 K (°C) over the same period. The discrepancy from the inferred 11.4 K may arise from local temperature differences between the airport and the city-centre university buildings. Nevertheless, as shown in Fig. S2), the observed range variation closely follows the general temperature trend recorded around Bristol Airport. The associated attenuation remains below 0.05 dB [4] for temperatures between -60 °C and 85 °C, which is negligible for this system, especially as the noise-reduction performance remains effective, and even more pronounced, under high transmission loss conditions.

Fibre movement: The system is inherently resilient to fibre-induced disturbances. The noise-suppression performance remains strong and becomes even more pronounced under high-loss and high-background conditions. Moreover, phase and polarization fluctuations do not impact the field trial, as the system does not rely on interferometric sensitivity. Consequently, bending losses, power variations, and external perturbations introduce no observable degradation during field trials.

V. COVERTNESS IN BRIGHTNESS

The covertness we are considering is the inability of the target to be able to distinguish whether the transmitter is on or off (i.e., are they being ranged). Our system is promising for this, as the intensity of the transmitter light at the target is less than the intensity of the solar background. A tighter constraint on covertness is that the number of photons that are collected at the target in a particular area is less than the fluctuation in photon number from the solar background in the same area. In this work, the average source power is 48 μW, producing an illumination spot of approximately 86.7 mm in diameter on the target building (WMB). This corresponds to an irradiance of 0.00813 W/m^2. By comparison, the solar background irradiance around 1550 nm wavelength is approximately 0.25 W/m^2/nm. Assuming the target selects the same 30 nm bandwidth used in our experiment, the effective solar background irradiance is 7.5 W/m^2, which is three orders of magnitude higher than the source. While our source power is not comparable to shot-noise fluctuations of the solar background,

environmental fluctuations in the solar background are expected to exceed 1% on short time scales and are often found to be over 10% [5]. Thus, our system satisfies this tighter covertness criterion, with the transmitted signal level more than 10 times lower than the background fluctuations.

VI. STATE-OF-THE-ART COMPARISON

A comprehensive comparison of state-of-the-art performance metrics is provided in Table 1, covering quantum rangefinding [1], chaotic quantum frequency conversion (chaotic-QFC, quantum-inspired) Light Detection and Ranging (LiDAR) [6], high-performance single-photon LiDAR systems [7–10], Gigahertz single-photon avalanche diode (GHz-SPAD) LiDAR [11], and frequency-modulated continuous-wave (FMCW) LiDAR technologies [12, 13]. Compared with quantum rangefinding demonstrations [1], the source brightness employed here is substantially higher yet remains within the covert-illumination limits established in the covert-analysis Section V. This enables long-distance operation without exceeding covert thresholds. By contrast, most classical LiDAR systems use optical powers > 1 mW, at least 20 times higher than in this work, and therefore operate outside the covert regime. Achieving ranges beyond 1 km with a low-reflective target typically requires either more than Watt illumination or highly sensitive, cryogenic detectors such as superconducting nanowire single-photon detectors (SNSPDs) in the reported systems. This is either not good for brightness covertness or compact detection system. It is worth noting that our approach can readily support longer distance through increased optical power or extended integration times.

Our experimental conditions also impose more stringent loss factors than those used in many previous field demonstrations. Measurements were performed using an older building with an irregular, low-reflectivity facade, introducing significant surface-scattering loss. In contrast, many long-range studies use high-reflectivity targets, where losses are dominated primarily by beam divergence. The receiver optics in our system consist of a compact 48 mm aperture, offering a portable and practical collection system for deployment. For detection, we use single-photon avalanche diodes (SPADs), which offer an effective balance of sensitivity, robustness, and cost. This contrasts with homodyne-based receivers, which, despite their sensitivity, are mechanically fragile and challenging to deploy outside the laboratory, and with SNSPDs, which are costly and require continuous cryogenic cooling.

The background illumination conditions in our measurements further represent a demanding and realistic outdoor scenario. As shown in Figs. 5(d), the target building is directly exposed to sunlight, resulting in a solar background level nearly three orders of magnitude higher than the transmitted power, as quantified in the covertness analysis in Section V. Many reported studies either simulate ambient noise in controlled laboratory settings or conduct measurements under standard daylight conditions without quantifying the background level. The results presented here therefore demonstrate system performance under overwhelmingly strong and realistic background noise levels encountered in practice.

In conclusion, our field trial employed an ultra-low-power (48 μW) source to detect a remote target directly illuminated by sunlight, representing an extremely high-background scenario. Even in these noise-dominated conditions, where the ambient background exceeded the signal by nearly three orders of magnitude, the system successfully recovered signal photons. These results highlight the practical robustness and exceptional noise tolerance of the approach, demonstrating its suitability for real-world deployment.

VII. SCALABILITY

Considering scalability to a larger number of channels, integrated photonic platforms [14, 15] represent a natural and practical path forward. They effectively address system complexity and calibration challenges while offering compact, robust, and cost-efficient solutions for multi-channel optical architectures. Once the chip design is finalized, the integrated configuration provides intrinsically stable alignment and straightforward operation. Detector requirements can be addressed by employing InGaAs detector arrays [9, 16] or by operating in the near-infrared or visible regions, where high-performance silicon detectors are readily available. High-precision optical filtering can be achieved using mature commercial dense wavelength-division multiplexing (DWDM) components [17, 18], which routinely support 96 channels with low crosstalk (adjacent channel isolation > 25 dB) and excellent spectral stability. Alternatively, integrated arrayed waveguide gratings offer a compact and robust solution for scalable wavelength multiplexing [19]. Finally, the field-programmable gate array (FPGA) we use is an intermediate model that is cost effective and can be used for up to 16 channels. To increase channel number, we can synchronise multiple of these together [20] or opt for a higher specification model, enabling parallel processing and straightforward system expansion.

Table 1 State-of-the-Art Performance Comparison for Quantum and Classical Lidar System

System	Avg. Tx Power	Detection Range	Detected Target	Collection Aperture	Detector Type	Background Noise
Quantum-inspired Rangefinding (This work)	48 µW	413 m	Building	48 mm	Single-mode-fibre (SMF) coupled SPAD	Direct solar irradiation on target (Background ≈ 103 × signal)
Quantum Rangefinding [1]	pW-scale	3 m (lab)	Corner-cube retroreflector	48 mm	Si-APD	LED-simulated
Chaotic-QFC LiDAR (quantum-inspired) [6]	50 mW	4 m (lab)	Indoor quadcopter	50.8 mm	Balanced Homodyne Detection	Simulated by amplified spontaneous emission (ASE) noise
SNSPD Single Photon LiDAR [7]	2.9 mW	1 km	Comms mast (metal frame)	254 mm	SMF-coupled SNSPD	Daylight
Satellite Single-Photon LiDAR [8]	15 W	935.9 km	Retroreflective satellite	280 mm	SMF-coupled SPAD	Night
Single Photon LiDAR with 64x64 SPAD Array [9]	2.5 W	5.7 km	Building	50 mm	64×64 InGaAs/InP SPAD array	Daylight
High-Repetition-Rate Single Photon LiDAR [10]	200 mW	340 m	DJI mini3	40 mm	Multi-mode-fibre (MMF) coupled SNSPD	Not stated
Airborne GHz-SPAD LiDAR [11]	107 mW	620 m	Ground	15.3 mm	MMF-coupled SPAD	Morning (Background ≈ 30× signal)
OPA-based FMCW LiDAR [12]	≤ 40 W	2 km	Building	350 mm	Free space InGaAs APD	Night
Long-range FMCW LiDAR [13]	1 W CW	3 km	Houses	20 mm	Coherent balanced photodiodes	Clear air

[1] S. Frick, A. McMillan, and J. Rarity, Quantum rangefinding, Opt. Express **28**, 37118 (2020).

[2] Data downloaded from `https://weatherspark.com/h/d/39587/2024/10/12/Historical-Weather-on-Saturday-October-12-2024-in-Bristol-United-Kingdom`.

[3] R. Slavík, G. Marra, E. N. Fokoua, N. Baddela, N. V. Wheeler, M. Petrovich, F. Poletti, and D. J. Richardson, Ultralow thermal sensitivity of phase and propagation delay in hollow core optical fibres, Sci. Rep. **5**, 15447 (2015).

[4] PDF available at `https://lewinb.net/posts/12_what_every_programmer_should_know_about_optical_fiber/PI-1470-AEN.pdf`.

[5] F. P. M. Kreuwel, W. H. Knap, L. R. Visser, W. G. J. H. M. van Sark, J. Vilà-Guerau de Arellano, and C. C. van Heerwaarden, Analysis of high frequency photovoltaic solar energy fluctuations, Sol. Energy **206**, 381 (2020).

[6] H. Liu, C. Qin, G. Papangelakis, M. L. Iu, and A. S. Helmy, Compact all-fiber quantum-inspired LiDAR with over 100 dB noise rejection and single photon sensitivity, Nat. Commun. **14**, 5344 (2023).

[7] A. McCarthy, G. G. Taylor, J. Garcia-Armenta, B. Korzh, D. V. Morozov, A. D. Beyer, R. M. Briggs, J. P. Allmaras, B. Bumble, M. Colangelo, D. Zhu, K. K. Berggren, M. D. Shaw, R. H. Hadfield, and G. S. Buller, High-resolution long-distance depth imaging LiDAR with ultra-low timing jitter superconducting nanowire single-photon detectors, Optica **12**, 168 (2025).

[8] Y. Li, W.-l. Ye, Z.-p. Li, M. Long, Z. Wu, Y. Cao, C.-z. Peng, and F. Xu, Compact single-photon LiDAR for satellite laser ranging, Opt. Express **33**, 40876 (2025).

[9] C. Tan, W. Kong, G. Huang, S. Jia, Q. Liu, Q. Han, J. Hou, R. Xue, S. Yu, and R. Shu, Development of a near-infrared single-photon 3D imaging LiDAR based on 64×64 ingaas/inp array detector and risley-prism scanner, Opt. Express **32**, 7426 (2024).

[10] W. Zhang, Z. Li, Y. Wang, X. Chen, D. Zhai, P. Zhang, H. Pan, and G. Wu, Sensitive micro UAV detection based on a high-repetition-rate single-photon LiDAR, Opt. Express **33**, 18102 (2025).

[11] G. Shen, T. Zheng, Z. Li, E. Wu, L. Yang, Y. Tao, C. Wang, and G. Wu, High-speed airborne single-photon LiDAR with ghz-gated single-photon detector at 1550 nm, Opt. Laser Technol. **141**, 107109 (2021).

[12] Y. Wu, S. Shao, Y. Li, X. Chen, D. Che, J. Chen, K. Du, R. Jiang, X. Huang, and D. Kan, Multi-beam optical phase array for long-range LiDAR and free-space data communication, Opt. Laser Technol. **151**, 108027 (2022).

[13] P. Feneyrou, A. Martin, D. Dolfi, and E. Payot, 3D imaging with large range dynamics and simultaneous accurate speed measurement, Appl. Opt. **63**, 5387 (2024).

[14] X. Xu, G. Ren, T. Feleppa, X. Liu, A. Boes, A. Mitchell, and A. J. Lowery, Self-calibrating programmable photonic integrated circuits, Nat. Photonics. **16**, 595 (2022).

[15] D. Pérez-López and L. Torrijos-Morán, Large-scale photonic processors and their applications, npj Nanophoton. **2**, 32 (2025).

[16] J. A. Dolphin, R. O. Scowen, L. M. Wells, D. J. Ellis, A. L. Lowe, B. Ramsay, J. I. Davies, A. J. Shields, T. K. Paraiso, and R. M. Stevenson, Hybrid-integrated ingaas/inp spad arrays for quantum communications, arXiv preprint arXiv:2509.05134 (2025).

[17] T. Ohara, H. Takara, T. Yamamoto, H. Masuda, T. Morioka, M. Abe, and H. Takahashi, Over-1000-channel ultradense wdm transmission with supercontinuum multicarrier source, J. Lightw. Technol. **24**, 2311 (2006).

[18] S. Singh and R. S. Kaler, Comparison of pre-, post- and symmetrical compensation for 96 channel dwdm system using pdcf and psmf, Optik **124**, 1808 (2013).

[19] Z. Wang, Z. Fang, Z. Liu, Y. Liang, J. Liu, J. Yu, T. Huang, Y. Zhou, H. Zhang, M. Wang, and Y. Cheng, On-chip arrayed waveguide grating fabricated on thin-film lithium niobate, Adv. Photon. Res. **5**, 2300228 (2024).

[20] Y. Xu, A. D. Rajagopala, N. Fruitwala, and G. Huang, Multi-FPGA synchronization and data communication for quantum control and measurement, arXiv preprint arXiv:2506.09856 (2025).
